# Do different male infertility factors impact embryological, cumulative pregnancy and neonatal outcomes in IVF/ICSI cycles? A retrospective cohort study

**DOI:** 10.1093/hropen/hoaf073

**Published:** 2025-11-25

**Authors:** Liu Jiang, Jiayin Zhou, Haoming Huang, Yan Li, Mingwei Lv, Yueping Zhou, Yuchen Gong, Xinyao Hu, Jie Li, Zhiqi Liao, Xiujuan Tan, Lei Jin, Kun Qian

**Affiliations:** Reproductive Medicine Centre, Tongji Hospital, Tongji Medical College, Huazhong University of Science and Technology, Wuhan, China; Reproductive Medicine Centre, Tongji Hospital, Tongji Medical College, Huazhong University of Science and Technology, Wuhan, China; Reproductive Medicine Centre, Tongji Hospital, Tongji Medical College, Huazhong University of Science and Technology, Wuhan, China; Reproductive Medicine Centre, Tongji Hospital, Tongji Medical College, Huazhong University of Science and Technology, Wuhan, China; Department of Gynaecology and Obstetrics, Tongji Hospital, Tongji Medical College, Huazhong University of Science and Technology, Wuhan, China; Reproductive Medicine Centre, Tongji Hospital, Tongji Medical College, Huazhong University of Science and Technology, Wuhan, China; Reproductive Medicine Centre, Tongji Hospital, Tongji Medical College, Huazhong University of Science and Technology, Wuhan, China; Reproductive Medicine Centre, Tongji Hospital, Tongji Medical College, Huazhong University of Science and Technology, Wuhan, China; Reproductive Medicine Centre, Tongji Hospital, Tongji Medical College, Huazhong University of Science and Technology, Wuhan, China; Cancer Biology Research Center (Key Laboratory of the Ministry of Education), Tongji Hospital, Tongji Medical College, Huazhong University of Science and Technology, Wuhan, China; Reproductive Medicine Centre, First Hospital of Wuhan, Tongji Medical College, Huazhong University of Science and Technology, Wuhan, China; Reproductive Medicine Centre, Tongji Hospital, Tongji Medical College, Huazhong University of Science and Technology, Wuhan, China; Reproductive Medicine Centre, Tongji Hospital, Tongji Medical College, Huazhong University of Science and Technology, Wuhan, China

**Keywords:** male infertility factor, clinical outcome, cumulative live birth, propensity score matching, neonatal outcome

## Abstract

**STUDY QUESTION:**

What are the impacts of different male infertility factors on embryological, cumulative pregnancy and neonatal outcomes of IVF/ICSI cycles?

**SUMMARY ANSWER:**

Some severe male infertility factors, i.e. severe oligoasthenozoospermia (OAT-S) and non-obstructive azoospermia (NOA), may be negatively associated with fertilization, embryo development, and cumulative live birth rates, but not with neonatal outcomes.

**WHAT IS KNOWN ALREADY:**

Previous studies examining the effect of male infertility factors on IVF/ICSI clinical outcomes have drawn contradictory conclusions, largely because the semen quality of male partners could fluctuate due to many factors, and there are many confounding factors from female partners.

**STUDY DESIGN, SIZE, DURATION:**

This retrospective cohort study involved 4714 males with various semen abnormalities and 10 283 males with normozoospermia whose partners underwent their first IVF/ICSI cycle between January 2018 and September 2022 in the reproductive medicine centre of a university hospital.

**PARTICIPANTS/MATERIALS, SETTING, METHODS:**

Only couples with infertility caused by fallopian tubal factors, male factors, or unknown reasons were included. The patients were divided into five different groups: normozoospermia (N), mild–moderate male factor (MMF), OAT-S, azoospermia-husband (Azoospermia-H), and azoospermia-donor (Azoospermia-D). The Azoospermia-H group was further divided into obstructive azoospermia (OA) and NOA. We compared rates of fertilization, embryo development, and cumulative pregnancy as well as neonatal outcomes. Reproductive and neonatal outcomes of men with various semen abnormalities were studied through propensity score matching (PSM) comparisons along with corresponding control groups (N) (with matching factors: female age, female BMI, male age, male BMI, ovarian stimulation protocol, number of oocytes obtained, and endometrial thickness). Fertilization outcomes were also compared and stratified by IVF or ICSI.

**MAIN RESULTS AND THE ROLE OF CHANCE:**

The mean female ages in the azoospermia, OAT-S, MMF, and N groups were 28.9, 29.4, 31.0, and 31.0 years old, respectively, which were similar between groups after PSM. The normal fertilization rates were significantly reduced in the OAT-S and Azoospermia-H groups compared with the control group in ICSI cycles (68.1% vs 71.5%, *P *= 0.001; 65.3% vs 72.4%, *P *< 0.001). The embryo utilization rates were also significantly decreased in the OAT-S and Azoospermia-H groups compared with controls in IVF/ICSI cycles (48.8% vs 57.3%, *P *< 0.001; 53.9% vs 58.1%, *P *= 0.001). Regarding pregnancy outcomes, the cumulative live birth rate in the OAT-S group was decreased (66.3% vs 74.5%, OR 0.68, 95% CI: 0.56–0.81). Among azoospermia cases, the NOA group exhibited a lower live birth rate (66.4% vs 75.8%, OR 0.63, 95% CI: 0.40–0.99), and an increased pregnancy loss rate (18.2% vs 9.4%, OR 2.15, 95% CI: 1.20–3.85) compared with the control group. No impact of male infertility factor on obstetrical/perinatal outcomes was observed. In IVF/ICSI cycles, reproductive and neonatal outcomes were similar between the MMF, Azoospermia-D, OA, and control groups.

**LIMITATIONS, REASONS FOR CAUTION:**

The main limitation of this study was the observational and retrospective design itself. Despite covariate adjustment, residual bias remained, and the single-centre cohort limited its generalizability.

**WIDER IMPLICATIONS OF THE FINDINGS:**

These findings offer new insights for the OAT-S and NOA groups for whom interventions before IVF/ICSI could be encouraged. Reassuringly, IVF/ICSI may be an effective and safe method for patients in the MMF, Azoospermia-D, and OA groups, avoiding additional medical treatments and associated burdens.

**STUDY FUNDING, COMPETING INTEREST(S):**

This study was supported by grants from the National Key Research and Development Plan Fund (No. 2018YFA0108400). The funders had no role in the study design, data collection or analysis, publication decision, or manuscript preparation. The authors declare that they have no competing interests.

**TRIAL REGISTRATION NUMBER:**

N/A.

WHAT DOES THIS MEAN FOR PATIENTS? Poor sperm quality is a major cause of infertility. In fact, around the world, sperm count and motility are getting worse over time, making this problem increasingly common. While embryo quality was once thought to be driven mainly by maternal factors, we now recognize the active role of sperm beyond fertilization. However, we do not fully understand how different types of sperm problems affect embryonic developmental potential, cumulative pregnancy chances, and neonatal outcomes. To address this, we analysed nearly 15 000 assisted reproduction cases.

We found pregnancy and neonatal outcomes similar to those for the normal semen group with some of types of male infertility, including mild to moderate semen abnormalities, abnormal sperm morphology, azoospermia (when semen contains no sperm) due to blocked sperm ducts, and azoospermia where donor sperm were used. For these patients, extra treatment before IVF may not be necessary, which could help reduce costs. By contrast, men with more severe sperm problems, such as very low sperm counts and motility and azoospermia with sperm production failure, had lower chances of success. For these patients, additional treatments or advanced sperm-selection methods may be helpful before IVF/ICSI. Reassuringly, none of these factors were linked to higher risks of pregnancy complications or birth defects.

## Introduction

Currently, 10–15% of couples are affected by infertility (Barbu *et al.*, 2021), and male infertility factors account for 30–50% of causes (Kasman *et al.*, 2021; Minhas *et al.*, 2021; de Ligny *et al.*, 2022), which impact ∼18 million men worldwide (Rimmer *et al.*, 2022). A systematic review and meta-regression analysis of 223 studies across six continents (1973–2018) documented a 51.6% decline in mean sperm concentration and a 62.3% decline in total sperm count among unselected men globally (Levine *et al.*, 2023). Male infertility is typically classified on the basis of medical history taking, physical examination, semen analysis, hormone testing, and genetic assessment into oligospermia, asthenospermia, teratospermia, non-obstructive azoospermia (NOA), and obstructive azoospermia (OA) (Schlegel *et al.*, 2021; Minhas *et al.*, 2025). ICSI has revolutionized severe male infertility management, enhancing embryo development and enabling biological paternity, particularly when integrated with testicular sperm extraction (Schlegel, 1999; Flannigan and Schlegel, 2019). This progress has coincided with a paradigm shift. Historically, embryonic compromise was attributed primarily to maternal factors, whereas emerging evidence has underscored the active role of sperm beyond fertilization (Vallet-Buisan *et al.*, 2023).

Despite these advances, the potential influence of different male infertility factors on pregnancy and neonatal outcomes following IVF or ICSI treatment, compared to couples with normozoospermia, remains insufficiently characterized. Several studies have explored the relationship between oligoasthenozoospermia and embryo quality or pregnancy outcomes from various aspects. Some reports have associated severe oligospermia impairments with compromised early embryonic development, manifesting as delayed cleavage rates and reduced cleavage-stage embryo quality (Borges *et al.*, 2024; Pellegrini *et al.*, 2024), alongside asthenozoospermia with diminished fertilization rates, poorer blastocyst quality, and lower live birth rates in ICSI cycles (Vogiatzi *et al.*, 2022). In contrast, the oocyte donation model showed that various semen abnormalities did not affect clinical or neonatal outcomes (Capelouto *et al.*, 2018; Cozzolino *et al.*, 2025). The prognostic implications of NOA are particularly contentious. NOA was associated with significantly lower fertilization rates (Mazzilli *et al.*, 2017; Grammatis *et al.*, 2023; Romano *et al.*, 2025) and blastocyst formation rates (Mazzilli *et al.*, 2017). One study reported a substantially lower live birth per transfer in NOA patients versus oligospermic counterparts (20.4% vs 30.3–35.4%), alongside increased miscarriage (11.8% vs 2.7–7%) and preterm birth rates (55.1% vs 16–46.8%) (Grammatis *et al.*, 2023). Yet another study found no significant difference in live birth rate across varying degrees of spermatogenic dysfunction, including NOA (Ping *et al.*, 2022). Regarding sperm morphology, research has revealed its predictive value for ICSI success (Villani *et al.*, 2022), yet paradoxically, patients with the most severe teratozoospermia (0% normal morphology) can achieve higher clinical pregnancy rates and better blastocyst quality than those with ≥5% normal morphology (French *et al.*, 2010).

The conclusions drawn from existing studies are limited in their robustness by the small sample size, heterogeneous outcome reporting, and absence of cumulative pregnancy outcomes. We therefore aimed to assess the impact of different male infertility factors on IVF/ICSI embryological, cumulative pregnancy and neonatal outcomes compared with those with normozoospermia. Two design challenges required attention: first, semen quality in male partners can vary due to multiple factors, including daily fluctuations (Liu *et al.*, 2022). Second, confounding factors related to the female partner’s baseline characteristics (particularly age) impact outcomes. To mitigate these issues, our design incorporated at least two semen examinations and employed propensity score matching (PSM) to balance key baseline variables.

## Materials and methods

### Study design and patients

**Figure 1.**
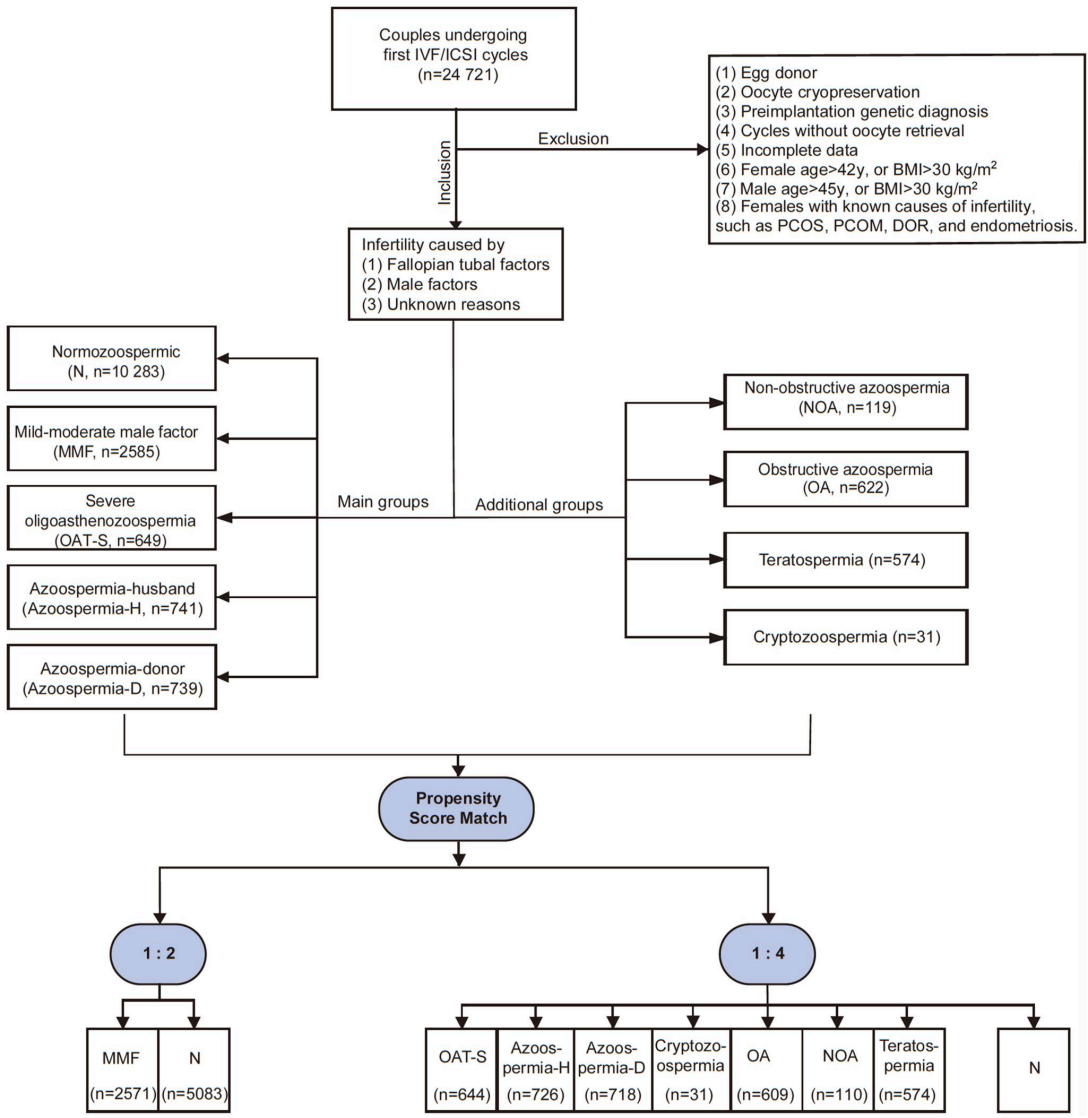
**Study population flowchart and group-matching strategy**. This flowchart illustrates the patient selection and group allocation for analysing the effects of male infertility factors on assisted reproduction outcomes. The male-factor infertility cohort was divided into eight groups: main groups (MMF, OAT-S, Azoospermia-H, Azoospermia-D) and additional groups (Cryptozoospermia, OA, NOA, and Teratospermia). PCOS, polycystic ovary syndrome; PCOM, polycystic ovarian morphology; DOR, diminished ovarian reserve; Azoospermia-D, Azoospermia and using donor sperm; Azoospermia-H, Azoospermia and using the husband’s sperm; OAT-S, severe oligoasthenozoospermia; MMF, mild–moderate male factor; N, normozoospermia; OA, obstructive azoospermia; NOA, non-obstructive azoospermia.

This retrospective cohort study was conducted at the Reproductive Medicine Centre, Tongji Hospital, Huazhong University of Science and Technology. Couples underwent their first IVF/ICSI cycles between January 2018 and September 2022, with follow-up continuing until September 2023. Only couples with infertility caused by fallopian tubal factors, male factors, or unknown reasons were included. Exclusion criteria were: (i) oocyte donors, (ii) oocyte cryopreservation, (iii) preimplantation genetic diagnosis, (iv) cycles without oocyte retrieval, (v) incomplete data, (vi) female age over 42 years old or BMI over 30 kg/m^2^, (vii) male age over 45 years old or BMI over 30 kg/m^2^, and (viii) females with known causes of infertility, such as polycystic ovary syndrome (PCOS), polycystic ovarian morphology (PCOM), diminished ovarian reserve (DOR), and endometriosis. Figure 1 shows the flowchart of patient inclusion and analysis.

### Ethical approval

This cohort study was approved by the Ethics Committee of Huazhong University of Science and Technology (approval number TJ-IRB20230921) following the Helsinki declaration and Strengthening the Reporting of Observational Studies in Epidemiology (STROBE) guidelines. Due to its retrospective nature with de-identified data, informed consent was waived.

### Main groups

Patients were categorized into five principal groups, covering all IVF/ICSI treatment cycles, as follows. (i) Normozoospermia (N) group: defined as sperm concentration ≥15 × 10^6^/ml and forward semen motility ≥32%. Both criteria were required simultaneously for inclusion, establishing this group as the reference baseline. (ii) Severe oligoasthenozoospermia (OAT-S) group: defined as sperm concentration <5 × 10^6^/ml and forward semen motility <10%. Subjects were assigned to this group only if both criteria were concurrently met, signifying severe semen deficiency. (iii) Mild–moderate male factor (MMF) group: encompassed subjects with semen parameters falling between the N and OAT-S group definitions. This included diverse presentations such as: concentration ≥15 × 10^6^/ml with forward semen motility between 10% and 32%, or motility ≥32% with concentration between 5 and 15 × 10^6^/ml. (iv) Azoospermia-husband (Azoospermia-H) group: comprised cycles utilizing sperm obtained via surgical retrieval for ICSI in cases of confirmed azoospermia, including NOA and OA. (v) Azoospermia-donor (Azoospermia-D) group: encompassed cycles where normal donor sperm were utilized for either IVF or ICSI.

### Additional groups

The following groups were analysed separately and distinctly from the primary groups for additional group analysis. (i) Teratozoospermia group: subjects were included if sperm morphology demonstrated >96% abnormal morphology. To minimize confounding factors (such as the effects of asthenospermia or oligospermia), this group was restricted to individuals concurrently exhibiting sperm concentration ≥15 × 10^6^/ml and forward sperm motility ≥32%. (ii) Cryptozoospermia group: subjects were classified under this heading if routine semen microscopy revealed no spermatozoa, but subsequent microscopic examination of the centrifuged pellet (2000 *g* × 15 min) identified the presence of sperm.

### Semen analysis

All male infertile patients included in the study underwent at least two routine semen analyses before their partner entered the IVF ovarian stimulation cycle, with an interval of no less than 1 week. Parameters assessment encompassed sperm concentration, forward sperm motility, and sperm morphology, adhering strictly to World Health Organisation (WHO, 2010) criteria. In addition, on the day of oocyte retrieval, all patients underwent semen assessments before sperm processing, evaluating sperm concentration and forward sperm motility.

The final patient group assignment was contingent upon concordance between the semen analysis results before the IVF stimulation cycle and the analysis performed on the day of oocyte retrieval. If results were discordant (e.g. pre-IVF findings suggested OAT-S criteria, while retrieval-day parameters qualified for MMF), the patient was allocated to the group representing the less severe category (e.g. assignment to MMF superseding OAT-S).

### Protocol for ovarian stimulation, fertilization, and embryo culture

All female patients were treated with gonadotropin-releasing hormone agonists (GnRH-a) or antagonist (GnRH-ant) protocols for ovarian stimulation (Xu *et al.*, 2018; Jiang *et al.*, 2019; Su *et al.*, 2022). In the GnRH-a protocol, the downregulation regimen included daily injections of 0.1 mg GnRH-a for 14 days starting from the mid-luteal phase, or 3.75 mg GnRH-a on the second day of menstruation. In the GnRH-ant protocol, cetopeptide (0.25 mg s.c., Merck Serono, Geneva, Switzerland) was injected daily when the average diameter of the dominant follicle was over 14 mm. Conventional fertilization protocols included IVF and ICSI based on the semen status on the day of oocyte retrieval. Following oocyte retrieval, all oocytes in standard IVF cycles were inseminated with 10 000 progressively motile spermatozoa after a 4-h incubation, whereas cases of severe oligospermia or previous fertilization failure were directed to ICSI. Surgical sperm retrieval methods of the Azoospermia-H group included OA with percutaneous epididymal sperm aspiration or testicular sperm aspiration and NOA by microscopic testicular sperm extraction (micro-TESE) (Song *et al.*, 2020). The laboratory procedure of embryo culture was reported previously (Jiang *et al.*, 2023).

### Definition of clinical outcomes

The primary outcome was the cumulative live birth rate, which was presented as the proportion of patients who achieved at least one live birth after multiple embryo transfers in the same oocyte retrieval cycle over 2 years. Only the first live birth resulting from any number of embryo transfers within a single oocyte retrieval cycle was considered; subsequent live births within the same cycle were not additionally counted towards the cumulative live birth rate. Live birth was defined as surviving babies delivered after 24 weeks. The secondary outcomes included embryologic, pregnancy, obstetric, and neonatal outcomes. The embryologic outcomes were defined according to the Vienna consensus (ESHRE Special Interest Group of Embryology and Alpha Scientists in Reproductive Medicine, 2017). The normal fertilization rate in IVF patients was defined as the percentage of 2PN embryos among the total number of oocytes obtained, while in ICSI cases, the normal fertilization rate was 2PN number divided by the number of mature oocytes. Top-quality embryos on Day 3 (D3) were defined as embryos from 2PN that developed at least seven cells of equal size with a fragmentation rate of no more than 10% (Gardner *et al.*, 2000). The embryo utilization index was the proportion of transferred or vitrified embryos and blastocysts relative to the total number of 2PNs. Clinical pregnancy was defined as the presence of a gestational sac in the uterus at 7 weeks of pregnancy. Pregnancy loss reflected miscarriage before 24 weeks of clinical pregnancy. Obstetric and neonatal complications (Cai *et al.*, 2021; Hu *et al.*, 2021) included preterm birth (<37 gestational weeks), macrosomia (birthweight >4000 g), low birth weight (birthweight <2500 g), gestational diabetes, hypertensive disorders of pregnancy, placenta previa, and foetal malformations.

### Statistical analysis

PSM was applied to select control patients using the ‘MatchIt’ package in R. Logistic regression models were used to calculate propensity scores based on baseline characteristics. Matching was performed without replacement using the nearest-neighbour method with a calliper width of 0.02. In the analysis of embryonic and clinical outcomes, the matching variables for all groups comprised female age, female BMI, male age, male BMI, ovarian stimulation protocol, number of oocytes retrieved, and endometrial thickness; for the Cryptozoospermia group specifically, the matching variables were simplified to female age, female BMI, and male age owing to the smaller sample sizes. For main groups and additional groups, a corresponding control group (N group) was matched at a 1:4 ratio, except for the MMF group, which was matched at a 1:2 ratio due to its larger sample size and limited availability of eligible controls.

For fertilization outcome analysis, matching variables included female age, female BMI, male age, male BMI, the ovarian stimulation protocol, and the number of oocytes retrieved. However, for the Cryptozoospermia group, the matching variables were limited to female age, female BMI, and male age. ICSI cycles in the N group were selected as controls for the OAT-S, Azoospermia-H, NOA, OA, teratospermia, and cryptozoospermia groups, while IVF cycles in the N group were used as controls for the MMF and Azoospermia-D groups. In this part of the analysis, a 1:1 matching ratio was applied.

Continuous variables were assessed for normality using the Kolmogorov–Smirnov and Shapiro–Wilk tests. Normally distributed variables were expressed as means±SDs, and non-normally distributed variables as medians (first quartile, third quartile). Categorical variables were presented as frequencies and percentages. Comparisons between groups were made using the Student’s *t*-test or the Mann–Whitney *U*-test for continuous variables, and the chi-squared test or Fisher’s exact test for categorical variables, depending on data distribution. Logistic or linear regression was applied to analyse embryological, clinical, and neonatal outcomes.

Sample size estimation was based on expected differences in live birth rates between groups (31% vs 36.2%; 31% vs 39.9%; and 31% vs 19.2% for the MMF, OAT-S, and Azoospermia-H groups versus controls, respectively) (Mazzilli *et al.*, 2017). Assuming a two-sided alpha level of 0.05 and a power of 80%, at least 1294 participants were required in the MMF group, 452 in the OAT-S group, and 162 in the Azoospermia-H group.

Statistical analyses were conducted using R software (version 4.3.1; R Foundation for Statistical Computing, Vienna, Austria) and SPSS software (version 26.0; SPSS Inc., Chicago, IL, USA). All statistical tests were two-sided, and *P *< 0.05 was considered statistically significant.

## Results

### Baseline characteristics before and after PSM

The patient selection and exclusion criteria are illustrated in the flowchart of Fig. 1, and 14 997 cycles fulfilled our inclusion criteria. Specifically, there were 2585 cycles for the MMF study group, 649 cycles for the OAT-S study group, 741 cycles for the Azoospermia-H study group, 739 cycles for the Azoospermia-D study group, and 10 283 cycles for the control group. The baseline and IVF/ICSI characteristics variations among different aetiological cohorts were assessed between the control group and the study groups (Supplementary Table S1). Results showed significant differences in maternal characteristics (such as age, antral follicle count, and proportion of primary infertility), paternal characteristics, and the number of oocytes retrieved across groups. The mean female ages in the Azoospermia-D, Azoospermia-H, OAT-S, MMF, and N groups were 28.9 ± 3.6, 29.0 ± 3.7, 29.4 ± 3.7, 31.0 ± 3.8, and 31.0 ± 3.6 years, respectively. The baseline variables are shown in Table 1 after PSM, indicating that most variables were similar between the study and control groups.

**Table 1.** Baseline characteristics after propensity score matching.

Variable	MMF	Control^a^	*P*	OAT-S	Control^a^	*P*	Azoospermia-H	Control^a^	*P*	Azoospermia-D	Control^a^	*P*
No. of cycles	2571	5083		644	2483		726	2745		718	2699	
**Maternal characteristic**												
Age (years)	31.0±3.8	31.0±3.6	0.90	29.4±3.6	29.4±3.5	0.80	29.1±3.6	29.3±3.5	0.12	29.1±3.7	29.2±3.4	0.31
Antral follicle count (n)	14.1±5.7	13.9±5.5	0.34	15.1±5.5	14.6±5.7	0.90	14.5±5.4	14.3±5.7	0.45	14.9±5.7	14.8±5.8	0.81
Baseline FSH (mIU/ml)	7.3±1.9	7.3±1.9	0.45	7.3±1.9	7.3±1.9	0.98	7.3±1.8	7.3±2.0	0.80	7.3±1.8	7.2±1.9	0.72
AMH (ng/ml)	4.8±3.3	4.7±3.3	0.66	5.4±3.3	5.1±3.6	0.11	5.2±3.5	5.0±3.5	0.15	5.5±3.7	5.5±3.8	0.58
BMI (kg/m^2^)	21.8±2.8	21.8±2.8	0.91	21.8±2.9	21.8±2.8	0.82	21.7±2.9	21.7±2.7	0.91	21.7±2.9	21.7±2.8	0.65
Duration of infertility (years)	3.3±2.3	3.2±2.3	0.08	3.2±2.4	3.0±2.0	0.20	2.9±2.3	3.0±1.9	0.31	3.4±2.4	3.3±2.4	0.24
**Paternal characteristic**												
Age (years)	32.7±4.3	32.8±4.1	0.64	31.4±4.1	31.4±4.0	0.90	30.1±4.2	31.1±3.9	0.29	30.9±3.9	31.1±3.9	0.33
BMI (kg/m^2^)	23.8±3.1	23.6±4.3	0.82	23.4±4.1	23.8±3.7	0.64	23.6±5.2	23.6±3.8	0.97	24.0±2.8	23.7±3.0	0.59
**IVF/ICSI characteristic**												
Ovarian stimulation protocol			0.64			0.79			0.60			0.85
Agonist	1669 (64.9%)	3327 (65.5%)		449 (69.7%)	1744 (70.2%)		451 (62.1%)	1676 (61.1%)		412 (57.4%)	1538 (57.0%)	
Antagonist	902 (35.1%)	1756 (34.5%)		195 (30.3%)	739 (29.8%)		275 (37.9%)	1069 (38.9%)		306 (42.6%)	1161 (43.0%)	
Gn duration (days)	10.2±1.8	10.2±1.8	0.46	10.3±1.8	10.3±1.8	>0.99	10.2±1.8	10 .1±1.8	0.30	10.0±1.8	10.1±1.9	0.83
Total dose of Gn (IU)	2289.8±815.5	2304.9±815.6	0.44	2249.7±824.0	2251.6±816.4	0.96	2253.2±794.5	2216.1±816.6	0.27	2211.0±788.0	2159.8±808.9	0.13
Estradiol on trigger day (pg/ml)	2777.2±1688.5	2747.6±1615.9	0.46	2867.7±1560.1	2807.9±1653.6	0.40	2896.0±1591.1	2854.3±1687.5	0.55	2930.4±1598.7	2985.9±1783.6	0.45
Endometrial thickness (mm)	11.8±2.6	11.8±2.7	0.52	12.2±2.6	12.1±2.7	0.25	12.1±2.4	12.0±2.7	0.53	12.2±2.6	12.1±2.8	0.36
Number of oocytes retrieved (n)	13.5±6.5	13.4±6.3	0.86	14.2±6.6	14.1±6.5	0.68	14.5±6.3	14.2±6.8	0.34	14.5±6.6	14.4±6.8	0.87

a PSM factors included female age, female BMI, male age, male BMI, COH protocol, number of oocytes retrieved, and endometrial thickness.

Data are presented as mean±SD or proportions (%). *P*-values were calculated using Student’s *t*-test for continuous variables and chi-square test for categorical variables.

PSM, propensity score matching; MMF, mild–moderate male factor; OAT-S, severe oligoasthenoteratozoospermia; Azoospermia-H, Azoospermia-husband; Azoospermia-D, Azoospermia-donor; FSH, follicle stimulating hormone; AMH, anti-Müllerian hormone; BMI, body mass index; Gn, gonadotropins.

### Fertilization and embryologic outcomes

As shown in Table 2, the normal cleavage rates between groups were similar. Except for the OAT-S group (44.3% vs 48.1%, β = −0.039, 95% CI: −0.067, −0.010), the other groups were similar to those of the control group in the D3 high-quality cleavage embryo rates. The blastocyst formation rate was significantly reduced in OAT-S (62.5% vs 68.8%, β = −0.076, 95% CI: −0.101, −0.051) and Azoospermia-H groups (62.4% vs 69.1%, β = −0.065, 95% CI: −0.089, −0.042) and slightly reduced in MMF group (68.1% vs 69.1%, β = −0.017, 95% CI: −0.031, −0.004) compared with the control group. The embryo utilization index was impaired in all groups (MMF 55.0% vs 57.1%, β = −0.014, 95% CI: −0.026, −0.003; OAT-S 48.8% vs 57.3%, β = −0.072, 95% CI: −0.093, −0.051; Azoospermia-H 53.9% vs 58.1%, β = −0.036, 95% CI: −0.056, −0.016; Azoospermia-D 56.0% vs 57.4%, β = −0.025, 95% CI: −0.045, −0.005). Furthermore, to compare the fertilization rates among these four groups, we additionally matched the fertilization method (IVF/ICSI) using a 1:1 PSM. The adjusted baseline characteristics are shown in Supplementary Table S2. The results indicated that after matching, the groups were similar to their respective control groups. Among the four main groups, the OAT-S group (68.1% vs 71.5%, β = −0.035, 95% CI: −0.056, −0.014) and the Azoospermia-H group (65.3% vs 72.4%, β = −0.071, 95% CI: −0.092, −0.050) showed significantly lower fertilization rates in ICSI cycles compared with the control group.

**Table 2.** Embryologic outcomes after propensity score matching.

Variable	MMF	**Control** ^a^	*β (95% CI)*	OAT-S	**Control** ^a^	*β (95% CI)*	Azoospermia-H	**Control** ^a^	*β (95% CI)*	Azoospermia-D	**Control** ^a^	*β (95% CI)*
No. of MII (n)	11 (8, 15)	11 (8, 15)	−0.087 (−0.352, 0.177)	12 (9, 16)	11 (8, 15)	0.107 (−0.379, 0.592)	12 (9, 16)	12 (8, 15)	0.028 (−0.443, 0.500)	12 (9, 17)	12 (9, 16)	0.079 (−0.424, 0.581)
	11.3±5.7	11.4±5.5		11.8±5.9	11.6±5.5		11.8±5.3	11.7±5.9		12.6±6.1	12.5±6.1	
No. of 2PN (n)	8 (6, 11)	8 (6, 11)	−0.069 (−0.282, 0.144)	9 (6, 12)	8 (6, 11)	−0.179 (−0.576, 0.219)	8 (5, 11)	8 (6, 12)	**−0.666 (−1.047, −0.285)**	9 (6, 12)	9 (6, 12)	−0.225 (−0.654, 0.144)
	8.1±4.6	8.2±4.5		8.1±4.9	8.3±4.5		7.7±4.3	8.4±4.7		8.7±4.7	9.0±4.9	
Normal fertilisation rate (%)**^b^**	63.5±19.3	63.9±19.3	−0.001 (−0.018, 0.015)	68.1±20.5	71.5±17.8	**−0.035 (−0.056, −0.014)**	65.3±21.1	72.4±18.4	**−0.071 (−0.092, −0.050)**	60.9±18.1	62.6±17.8	−0.019 (−0.039, 0.001)
No. of normal cleavage embryos (n)	8 (6, 11)	8 (5, 11)	−0.167 (−0.384, 0.051)	8 (6, 12)	8 (6, 11)	**−0.491 (−0.825, −0.014)**	8 (5, 11)	8 (5, 11)	**−0.852 (−1.238, −0.466)**	8 (6, 11)	9 (6, 12)	−0.325 (−0.737, 0.088)
	8.2±4.6	8.3±4.6		8.1±4.8	8.5±4.6		7.7±4.3	8.6±4.8		8.9±4.9	9.2±5.0	
Normal cleavage rate (%)	98.2±5.7	97.7±6.4	0.002 (−0.001, 0.005)	97.5±5.8	97.7±6.4	−0.002 (−0.007, 0.004)	98.1±5.3	97.7±6.00	0.004 (−0.001, 0.009)	97.9±6.3	97.9±6.4	−0.001 (−0.007, 0.005)
No. of D3 high-quality cleavage embryos (n)	4 (2, 6)	4 (2, 6)	−0.059 (−0.228, 0.111)	4 (2, 6)	4 (2, 6)	−0.189 (−0.534, 0.156)	4 (2, 6)	4 (2, 6)	−0.255 (−0.534, 0.083)	4 (2, 6)	4 (2, 6)	−0.261 (−0.598, 0.075)
	4.0±3.1	4.1±3.1		3.9±3.3	4.1±3.2		3.9±3.1	4.1±3.2		4.2±3.2	4.5±3.4	
D3 high-quality cleavage embryo rate (%)	48.1±24.7	48.4±25.3	0.002 (−0.012, 0.017)	44.3±25.6	48.1±25.4	**−0.039 (−0.067, −0.010)**	47.0±25.5	47.6±25.3	−0.009 (−0.034, 0.017)	46.6±24.5	48.6±25.1	−0.017 (−0.043, 0.009)
No. of total cryopreserved blastocyst (n)	3 (1, 5)	3 (2, 5)	**−0.167 (−0.309, −0.025)**	3 (1, 5)	3 (2, 6)	**−0.720 (−0.981, −0.460)**	3 (1, 5)	3 (2, 6)	**−0.535 (−0.791, −0.279)**	3 (2, 6)	4 (2, 6)	−0.253 (−0.522, 0.016)
	3.2±3.0	3.3±3.0		2.7±2.8	3.5±3.1		3.0±2.8	3.5±3.2		3.6±3.2	3.8±3.3	
Blastocyst formation rate (%)	68.1±26.9	69.1±27.0	**−0.017 (−0.031, −0.004)**	62.5±28.3	68.8±27.1	**−0.076 (−0.101, −0.051)**	62.4±30.3	69.1±26.7	**−0.065 (−0.089, −0.042)**	68.5±26.8	70.6±26.2	−0.023 (−0.045, 0.000)
Viable blastocyst formation rate (%)	46.3±26.6	47.8±26.4	**−0.016 (−0.029, −0.003)**	41.5±26.4	48.1±26.0	**−0.076 (−0.100, −0.052)**	43.8±28.4	49.0±26.7	**−0.038 (−0.062, −0.015)**	46.0±26.1	49.2±26.2	**−0.035 (−0.057, −0.013)**
No. of available embryos (n)	4 (3, 6)	4 (3, 6)	**−0.158 (−0.290, −0.026)**	4 (3, 6)	4 (3, 7)	**−0.737 (−0.979, −0.495)**	4 (2, 6)	4 (3, 7)	**−0.549 (−0.786, −0.311)**	4 (3, 7)	5 (3, 7)	−0.230 (−0.480, 0.020)
	4.3±2.8	4.4±2.8		3.8±2.6	4.5±2.8		4.0±2.7	4.6±3.0		4.61±3.0	4.8±3.1	
Embryo utilisation index (%)	55.0±21.5	57.1±21.88	**−0.014 (−0.026, −0.003)**	48.8±22.5	57.3±21.4	**−0.072 (−0.093, −0.051)**	53.9±22.8	58.1±22.3	**−0.036 (−0.056, −0.016)**	56.0±22.2	57.4±22.1	**−0.025 (−0.045, −0.005)**

a PSM factors included female age, female BMI, male age, male BMI, COH protocol, number of oocytes retrieved, and endometrial thickness.

b Baseline characteristics of patients undergoing the same fertilisation method (IVF or ICSI) after 1:1 PSM are shown in Supplementary Table S2. Matching factors included female age, female BMI, male age, male BMI, COH protocol, and number of oocytes retrieved.

Data are presented as mean±SD or median (Q25, Q75). Data were compared between the study and the control group using the univariate linear regression analysis. Bold values indicate statistically significant differences.

PSM, propensity score matching; MMF, mild–moderate male factor; OAT-S, severe oligoasthenoteratozoospermia; Azoospermia-H, Azoospermia-husband; Azoospermia-D, Azoospermia-donor; β (95% CI), β-coefficient with 95% CI; D3, Day 3; MII, metaphase II; PN, pronucleus.

### Pregnancy and neonatal outcomes

Cumulative pregnancy outcomes for MMF, OAT-S, Azoospermia-H, and Azoospermia-D are shown in Table 3. In the OAT-S group, the clinical pregnancy rate (74.1% vs 81.4%, OR 0.65, 95% CI: 0.53–0.80) and live birth rate (66.3% vs 74.5%, OR 0.68, 95% CI: 0.56–0.81) were reduced. For MMF, Azoospermia-H and Azoospermia-D, pregnancy outcomes, including clinical pregnancy, ectopic pregnancy, pregnancy loss, and live birth, were similar to those in the control group. Reassuringly, no significant differences were found in neonatal outcomes in the male infertility groups (Table 4). All groups maintained similar obstetric safety profiles, with no increased risks of preterm birth or congenital anomalies.

**Table 3.** Pregnancy outcomes.

	MMF	**Control** ^a^	OR (95% CI)	OAT-S	**Control** ^a^	OR (95% CI)	Azoospermia-H	**Control** ^a^	OR (95% CI)	Azoospermia-D	**Control** ^a^	OR (95% CI)
Cumulative clinical pregnancy (n)	2028 (78.9%)	4051 (79.7%)	0.95 (0.85,1.07)	477 (74.1%)	2022 (81.4%)	**0.65 (0.53,0.80)**	576 (79.3%)	2195 (80.0%)	0.96 (0.79,1.18)	592 (82.5%)	2224 (82.4%)	1.01 (0.81,1.25)
Ectopic pregnancy (n)	19 (0.7%)	45 (0.9%)	0.83 (0.49,1.43)	2 (0.3%)	21 (0.8%)	0.37 (0.09,1.56)	2 (0.3%)	31 (1.1%)	0.24 (0.06,1.01)	1 (0.1%)	25 (0.9%)	0.15 (0.02,1.10)
Pregnancy loss (n)	303 (11.8%)	668 (13.1%)	0.88 (0.76,1.02)	87 (13.5%)	303 (12.2%)	1.12 (0.87,1.45)	97 (13.4%)	332 (12.1%)	1.12 (0.88,1.43)	78 (10.9%)	351 (13.0%)	0.82 (0.63,1.06)
Cumulative live births (n)	1842 (71.6%)	3645 (71.7%)	0.99 (0.90,1.11)	427 (66.3%)	1849 (74.5%)	**0.68 (0.56,0.81)**	535 (73.7%)	2014 (73.4%)	1.02 (0.84,1.22)	554 (77.2%)	2018 (74.8%)	1.14 (0.94,1.39)

a PSM factors included female age, female BMI, male age, male BMI, COH protocol, number of oocytes retrieved, and endometrial thickness.

Data are presented as proportions (%). Odds ratio (OR) and 95% CIs are based on the univariate logistic regression analysis. Bold values indicate statistically significant differences.

MMF, mild–moderate male factor; OAT-S, severe oligoasthenoteratozoospermia; Azoospermia-H, Azoospermia-husband; Azoospermia-D, Azoospermia-donor; PSM, propensity score matching.

**Table 4.** Obstetric and neonatal outcomes.

Variable	MMF	**Control** ^a^	OR (95% CI) or β (95% CI)	OAT-S	**Control** ^a^	OR (95% CI) or β (95% CI)	Azoospermia-H	**Control** ^a^	OR (95% CI) or β (95% CI)	Azoospermia-D	**Control** ^a^	OR (95% CI) or β (95% CI)
Gestational age (days)	255±13	255±11	0.24 (−0.41,0.91)	255±9	255±10	0.80 (−0.47,2.08)	256±15	253±14	0.95 (−0.17,2.06)	255±16	250±14	1.39 (0.24,2.54)
Multiple births (twins)	105 (5.7%)	226 (6.2%)	0.91 (0.72,1.16)	25 (5.9%)	127 (6.9%)	0.84 (0.54,1.31)	28 (5.2%)	128 (6.4%)	0.81 (0.53,1.24)	36 (6.5%)	148 (7.3%)	0.88 (0.60,1.28)
Neonatal birthweight of singletons (g)	2500±600	2515±500	−7.67 (−36.71,21.38)	2600±520	2500±500	−9.47 (−62.79,43.86)	2500±915	2525±400	−19.44 (−69.05,30.18)	2500±700	2550±600	4.19 (−44.94,53.32)
Neonatal birthweight of twins (means, g)	2475±665	2500±450	−64.65 (−161.08,31.779)	2525±345	2490±425	4.90 (−175.26,185.05)	2475±731	2450±450	−92.49 (−249.37,64.38)	2450±725	2450±500	−80.86 (−248.73,87.01)
**Singleton**												
Preterm birth	131/1737 (7.5%)	274/3419 (8.0%)	0.94 (0.75,1.16)	28/402 (7.0%)	122/1722 (7.1%)	0.98 (0.64,1.50)	33/507 (6.5%)	127/1886 (6.7%)	0.96 (0.65,1.43)	27/518 (5.2%)	140/1870 (7.5%)	0.68 (0.45,1.04)
Hypertensive disorders of pregnancy	40/1737 (2.3%)	80/3419 (2.3%)	0.98 (0.67,1.44)	7/402 (1.7%)	30/1722 (1.7%)	0.99 (0.44,2.29)	8/507 (1.6%)	43/1886 (2.3%)	0.69 (0.32,1.47)	5/518 (1.0%)	30/1870 (1.6%)	0.60 (0.23,1.55)
Gestational diabetes mellitus	53/1737 (3.1%)	93/3419 (2.7%)	1.13 (0.80,1.59)	9/402 (2.2%)	44/1722 (2.6%)	0.87 (0.42,1.80)	9/507 (1.8%)	47/1886 (2.5%)	0.71 (0.34,1.45)	16/518 (3.1%)	51/1870 (2.7%)	1.14 (0.64,2.01)
Placenta previa	34/1737 (2.0%)	72/3419 (2.1%)	0.93 (0.62,1.40)	9/402 (2.2%)	39/1722 (2.3%)	0.99 (0.48,2.06)	14/507 (2.8%)	44/1886 (2.3%)	1.19 (0.65,2.19)	12/518 (2.3%)	48/1870 (2.6%)	0.90 (0.48,1.71)
Foetal malformation	32/1737 (1.8%)	41/3419 (1.2%)	1.55 (0.97,2.46)	3/402 (0.7%)	19/1722 (1.1%)	0.67 (0.20,2.29)	5/507 (1.0%)	21/1886 (1.1%)	0.89 (0.33,2.36)	5/518 (1.0%)	27/1870 (1.4%)	0.67 (0.26,1.74)
Placenta abruption	0/1737 (0.0%)	2/3419 (0.1%)	NA	2/402 (0.5%)	1/1722 (0.1%)	8.61 (0.78,95.13)	1/507 (0.2%)	2/1886 (0.1%)	1.86 (0.17,20.57)	1/518 (0.2%)	1/1870 (0.1%)	3.62 (0.23,57.90)
Macrosomia >4000 *g*	75/1737 (4.3%)	172/3419 (5.0%)	0.85 (0.65,1.12)	16/402 (4.0%)	68/1722 (3.9%)	1.01 (0.58,1.76)	26/507 (5.1%)	93/1886 (4.9%)	1.04 (0.67,1.63)	17/518 (3.3%)	91/1870 (4.9%)	0.66 (0.39,1.12)
Low birthweight <2500*g*	61/1737 (3.5%)	155/3419 (4.5%)	0.77 (0.57,1.04)	17/402 (4.2%)	63/1722 (3.7%)	1.16 (0.67,2.01)	23/507 (4.5%)	74/1886 (3.9%)	1.16 (0.72,1.88)	18/518 (3.5%)	76/1870 (4.1%)	0.85 (0.50,1.43)

a Propensity score matching factors included female age, female BMI, male age, male BMI, ovarian stimulation protocol, number of oocytes retrieved, and endometrial thickness.

Data are presented as mean±SD or proportions (%). Data were compared between the study and the control group using the univariate logistic or linear regression analysis.

NA, not applicable (no cases, therefore odds ratio not estimable); MMF, mild–moderate male factor; OAT-S, severe oligoasthenoteratozoospermia; Azoospermia-H, Azoospermia-husband; Azoospermia-D, Azoospermia-donor; β (95% CI), β-coefficient with 95% CI.

### Analysis of additional groups

In addition, the pregnancy outcomes of the NOA, OA, Teratospermia, and Cryptozoospermia groups are summarized in Supplementary Table S3. The high-quality D3 cleavage embryo rate was significantly reduced in the NOA group compared with the control group (40.3% vs 49.2%, β = −0.089, 95% CI: −0.154, −0.025). Furthermore, both the NOA group (57.8% vs 68.9%, β = −0.111, 95% CI: −0.174, −0.048) and the OA group (62.5% vs 68.4%, β = −0.059, 95% CI: −0.084, −0.033) exhibited a decreased blastocyst formation rate. Additionally, the OA group showed a decline in the embryo utilization index (54.2% vs 57.6%, β = −0.034, 95% CI: −0.056, −0.013). However, no significant differences in embryological outcomes were observed between the teratospermia and cryptozoospermia groups and their respective control groups, with both showing similar results. Supplementary Table S4 presents the analysis of cumulative pregnancy outcomes in additional groups (NOA, OA, teratospermia, and cryptozoospermia). The NOA group exhibited impairments with a (non-significant) lower clinical pregnancy rate (74.5% vs 80.8%, OR 0.70, 95% CI: 0.43–1.14), diminished live birth rate (66.4% vs 75.8%, OR 0.63, 95% CI: 0.40–0.99), and increased pregnancy loss (18.2% vs 9.4%, OR 2.15, 95% CI: 1.20–3.85) compared with the control group. Cryptozoospermic cases showed a marked reduction in clinical pregnancy rates (61.3% vs 81.0%, OR 0.37, 95% CI: 0.16–0.87) and a (non-significant) decline in live birth rate (61.3% vs 70.2%, OR 0.67, 95% CI: 0.30–1.52). OA and teratospermia patients had pregnancy outcomes similar to the control group.

## Discussion

This large retrospective matched cohort study investigated the impact of MMF, OAT-S, Azoospermia-H, and Azoospermia-D on embryo quality, pregnancy, and neonatal outcomes in couples undergoing IVF/ICSI treatment. The multiple core reproductive outcomes were reported in accordance with the latest international consensus for male infertility trials (Rimmer *et al.*, 2025a,b). Our results showed no significant differences in the cumulative pregnancy or neonatal outcomes in the MMF, Azoospermia-H, and Azoospermia-D groups compared with the normozoospermia group. However, compared with the control group, the OAT-S study group had poorer fertilization, embryo development, and pregnancy outcomes. We then conducted additional analyses, separating NOA, OA, teratospermia, and cryptozoospermia. Compared with the control group, both the NOA and cryptozoospermia groups demonstrated significantly reduced fertilization, embryo development and/or pregnancy outcomes. The OA group showed reduced fertilization and blastocyst development outcomes, with no significant differences in pregnancy outcomes. The teratospermia group exhibited no significant differences in fertilization, embryo development, or pregnancy outcomes. No male infertility factor was found to impact obstetric or perinatal outcomes.

Previously, issues related to fertilization and embryonic development were often attributed to maternal factors alone. However, recent evidence suggests that paternal factors play a significant regulatory role in this process. Our study revealed that severe male infertility factors, including OAT-S, NOA, OA, and cryptozoospermia, exhibited a markedly increased risk of fertilization failure and impaired embryonic development, compared with patients presenting with normozoospermia. These findings are consistent with previous studies. A large-scale study involving 1090 couples and 1219 preimplantation genetic testing (PGT) cycles also showed elevated failure rates at the pre-blastocyst stage in the OA, NOA, and OAT-S groups (Mazzilli *et al.*, 2017). Among various semen parameters, sperm motility, in particular, forward motility, shows a strong positive correlation with both fertilization success (Villani *et al.*, 2022; Vogiatzi *et al.*, 2022) and high-quality embryo formation (Vogiatzi *et al.*, 2022). Meanwhile, the inhibitory effect of severe oligospermia on the fertilization process has been validated by multiple studies (Mazzilli *et al.*, 2017; Bartolacci *et al.*, 2018; Borges *et al.*, 2024; Cozzolino *et al.*, 2025). Taken together, these findings reveal that, although IVF/ICSI technology can bypass certain natural fertilization barriers, it cannot eliminate the profound regulatory effects of paternal factors, especially in patients with severe male infertility factors, on embryo development. While the underlying mechanisms remain incompletely understood, one plausible explanation involves oxidative stress-induced DNA fragmentation (DFI) (Dorostghoal *et al.*, 2017), as decreased motility is closely linked to compromised sperm DNA integrity (Stavros *et al.*, 2024). High DFI compromises genomic stability and may impair critical processes such as imprinting, gene maintenance, and DNA methylation reprogramming, leading to a significant deterioration in embryo quality (Simon *et al.*, 2011; Kim *et al.*, 2019; Ribas-Maynou *et al.*, 2021, 2022). Moreover, a meta-analysis of 10 clinical trials demonstrated that men with abnormal semen parameters had significantly more mitochondrial DNA in sperm cells than those with normozoospermia (Popova *et al.*, 2022). Similar conclusions were reached in a study of 5739 donor specimens (Sun *et al.*, 2022). Furthermore, a higher sperm mitochondrial DNA copy number and deletion rates in sperm are associated with lower fertilization rates, lower rates of high-grade embryos on Day 3, and poorer quality of transferred blastocysts (Wu *et al.*, 2019).

Although we found that fertilization and embryological outcomes were impaired in OAT-S, NOA, OA, and cryptozoospermia groups, whether these factors adversely affect cumulative pregnancy outcomes has been less clear. Our findings indicate that pregnancy outcomes in the MMF, OA, and teratospermia groups are similar to those in the normozoospermia group, which is consistent with previous studies (French *et al.*, 2010; Hayon *et al.*, 2021; Ping *et al.*, 2022; Cozzolino *et al.*, 2025). However, we observed a significant reduction in cumulative pregnancy outcomes in the OAT-S and NOA groups compared to those with normozoospermia. Although similar clinical studies are limited, there is some indirect evidence supporting our conclusions. Most studies have categorized semen parameters simply as normal or abnormal based on WHO reference ranges, often reporting similar clinical outcomes (Capelouto *et al.*, 2018; Mariappen *et al.*, 2018; Pastuszak *et al.*, 2019). Studies using continuous variables (Santi *et al.*, 2023) or quartiles (DeVilbiss *et al.*, 2022) to stratify semen parameters have shown an association between sperm motility/morphology and live birth rates, suggesting that stratifying patients based on the severity of semen abnormalities may offer greater clinical insight. We further infer that, when sperm quality declines below a certain threshold, it may compromise early embryonic development potential, and ICSI may be insufficient to overcome such profound deficiencies. This offers new insights for OAT-S patients: proactive pre-ART interventions should be considered to improve semen parameters. Regarding inconsistent literature reporting no effect of severe sperm concentration reduction on embryo euploidy or live birth rates, the differences may stem from: (i) differences in study design, i.e. the use of oocyte donation models and PGT models (Mazzilli *et al.*, 2017; Cozzolino *et al.*, 2025); and (ii) patient definition bias, as our study rigorously identified OAT-S as concurrent severe reductions in both motility and density across multiple semen analyses, which more precisely identified individuals with severely compromised sperm quality compared with simply ‘low concentration’. The total motile sperm count provided a more accurate prediction of spontaneous ongoing pregnancy rates than the WHO sperm classification system (Hamilton *et al.*, 2015). The effect of NOA on live birth rate is the most controversial, with some studies showing a significant decrease in live birth rate (Grammatis *et al.*, 2023) and some showing no difference (Ping *et al.*, 2022; Romano *et al.*, 2025), which may be related to study design, PGT-A application, sample size, cumulative outcome, confounding factors from female characteristics, and differences in micro-TESE techniques.

In addition, concerns have been raised about the safety of treatment and neonatal outcomes for couples undergoing IVF/ICSI due to male infertility factors. On the one hand, studies suggest a higher rate of chromosomal abnormalities in patients with severe male infertility factors (Kuroda *et al.*, 2020). Therefore, there is a theoretical concern that these potential genetic abnormalities may affect treatment outcomes. Nevertheless, our analysis found no significant differences in obstetric and neonatal outcomes (such as preterm birth and low birth weight) among patients with different male infertility factors. On the other hand, regarding the use of donor sperm, our results support its clinical efficacy and safety, without evident adverse effects on perinatal outcomes. For instance, a large-scale analysis, involving 134 710 fresh autologous ART cycles, revealed no significant disparities in miscarriage, preterm birth, or low birth weight rates between cycles employing donor or partner sperm (Yu *et al.*, 2018). These favourable outcomes may be attributed to high-quality sperm from optimally selected donors combined with advancements in cryopreservation and thawing techniques, maintaining clinical outcomes post-freezing (Huang *et al.*, 2019). It is also important to acknowledge that the relatively small sample size in our study may partly explain the similar neonatal outcomes between groups. Even so, our findings add to the limited amount of literature on perinatal outcomes, contributing supportive data to the ongoing discussion regarding the safety of ART in the context of severe male factor infertility.

The results of this study are of great significance to patient consultation and management in reproductive medicine. Our findings indicate that pregnancy and neonatal outcomes in patients with MMF, OA, and teratospermia are similar to those in patients with normozoospermia. Clinicians may therefore reassure patients that these specific male infertility factors do not appear to significantly impact reproductive outcomes in the context of current ART practice, thus suggesting that radical medical interventions may be unnecessary. This, in turn, can help avoid excessive financial burden and the psychological stress often associated with overtreatment. In contrast, setting realistic expectations is crucial for patients with OAT-S, cryptozoospermia, or NOA. These couples should be informed that they may face a risk of suboptimal fertilization, poorer embryo outcomes, and reduced cumulative live birth rates per cycle. For these couples, clinicians can discuss the following strategies: interventions for male infertility before ART, such as exercise or antioxidant supplements, and the use of advanced sperm selection techniques such as microfluidics or physiological ICSI (PICSI) to potentially improve prognosis (Hart *et al.*, 2025). For example, a meta-analysis has shown that antioxidants such as L-carnitine, vitamin E plus selenium/zinc, theobromine, vitamin E combined with coenzyme Q10, or folic acid may improve specific semen parameters in idiopathic oligoasthenozoospermia (Zafar *et al.*, 2023; Barbonetti *et al.*, 2024). Notably, the reassuring outcomes observed in donor sperm cycles underscore its value as a safe and effective option, particularly when surgical sperm retrieval fails or is not feasible. This evidence can support more informed decision-making regarding the choice between surgical extraction and alternative pathways for NOA patients. Overall, these findings help fertility experts develop personalized treatment plans based on the severity and type of male infertility, ultimately improving the scientific nature of patient management and decision-making.

The key strengths of this study are as follows. (i) We used a standardized core outcome set for male infertility trials, aligned with international consensus (Rimmer *et al.*, 2025a,b), to minimize reporting bias and enable future meta-analyses. (ii) We matched various female factors associated with the success of ART by PSM methods. Earlier ART intervention for severe male factor infertility leads to treatment at younger female ages (Mazzilli *et al.*, 2017). (iii) Potential influences of male age and BMI were also taken into account, with advanced paternal age specifically linked to adverse reproductive outcomes, including elevated DNA mutations and serving as an auxiliary fertility marker (McPherson *et al.*, 2018; Oldereid *et al.*, 2018; Chen *et al.*, 2021). (iv) Repeated semen analyses accounted for short-term variability when classifying normozoospermia, MMF, and OAT-S groups, enhancing assignment accuracy. However, the study has several limitations. (i) The study design was a single-centre, observational, retrospective study; nevertheless, its large sample size and the application of PSM to a wide range of potential confounding variables increase the reliability of the conclusions to some extent. (ii) Data on the sperm DNA fragmentation index were not feasible in the database, which limits interpretation of the potential underlying mechanisms driving the observed differences in outcomes. (iii) As the study was based on the cumulative live birth rate from complete oocyte retrieval cycles (including fresh and frozen embryos), confounding factors such as endometrial preparation protocols and culture conditions were not adjusted for. (iv) Teratozoospermia was defined based on semen analysis conducted before ovarian stimulation. A rigorous assessment and comparison of sperm morphology on the day of oocyte retrieval was not feasible, as morphological staining would have rendered the semen unsuitable for fertilization. (v) Long-term reproductive safety and the health of offspring conceived from severe male infertility factor cases remain underexplored.

## Conclusions

In summary, we observed that severe male infertility factors (OAT-S, NOA, and cryptozoospermia) may be negatively associated with fertilization, embryo development, and cumulative live birth rate, while those with MMF, OA, teratospermia, and azoospermia but using donor sperm groups exhibited similar pregnancy outcomes to control groups. Importantly, no significant differences in obstetric or perinatal outcomes were observed across the various male infertility categories, providing additional reassurance regarding the safety of ART in these populations. This study offers a comprehensive assessment of how the severity and aetiology of male infertility affect ART outcomes, thereby supporting more individualized clinical counselling and informing future research priorities.

## Supplementary Material

hoaf073_Supplementary_Data

## Data Availability

The data underlying this article are available within the article and its online supplementary material.
