# Supplementary material for: Do different male infertility factors impact embryological, cumulative pregnancy and neonatal outcomes in IVF/ICSI cycles? A retrospective cohort study
Source: Hum Reprod Open. 2025 Nov 25;2025(4):hoaf073. doi: 10.1093/hropen/hoaf073 (PMC12701804; doi:10.1093/hropen/hoaf073)
Supplement: hoaf073_Supplementary_Data [file hoaf073_supplementary_data.docx]

**Supplementary Table S1.** Baseline characteristics and IVF/ICSI characteristics before PSM.

| **Variable** | **MMF** | **Control ^a^** | ***P*** | **OAT-S** | **Control ^a^** | ***P*** | **Azoospermia-H** | **Control ^a^** | ***P*** | **Azoospermia-D** | **Control ^a^** | ***P*** |
| --- | --- | --- | --- | --- | --- | --- | --- | --- | --- | --- | --- | --- |
| No. of cycles | 2585 | 10 283 |  | 649 | 10 283 |  | 741 | 10 283 |  | 739 | 10 283 |  |
| **Maternal characteristic** |  |  |  |  |  |  |  |  |  |  |  |  |
| Age (y) | 31.0±3.8 | 31.0±3.6 | 0.38 | 29.4±3.7 | 31.0±3.6 | **<0.001** | 29.0±3.7 | 31.0±3.6 | **<0.001** | 28.9±3.6 | 31.0±3.6 | **<0.001** |
| Antral follicle count (n) | 14.1±5.7 | 13.8±5.6 | **0.04** | 15.2±5.5 | 13.8±5.6 | **<0.001** | 14.5±5.4 | 13.8±5.6 | **0.001** | 15.0±5.7 | 13.8±5.6 | **<0.001** |
| Baseline FSH (mIU/ml) | 7.3±1.9 | 7.4±2.0 | 0.07 | 7.3±1.9 | 7.4±2.0 | 0.23 | 7.3±1.8 | 7.4±2.0 | 0.24 | 7.3±1.8 | 7.4±2.0 | 0.18 |
| AMH (ng/ml) | 4.8±3.3 | 4.7±3.3 | 0.29 | 5.4±3.3 | 4.7±3.3 | **<0.001** | 5.2±3.5 | 4.7±3.3 | **<0.001** | 5.6±3.8 | 4.7±3.3 | **<0.001** |
| BMI (kg/m^2^) | 21.8±2.8 | 21.8±2.8 | 0.95 | 21.8±2.9 | 21.8±2.8 | 0.95 | 21.7±2.9 | 21.8±2.8 | 0.33 | 21.7±2.9 | 21.8±2.8 | 0.24 |
| Primary infertility, No.(%) | 1887 (73.0%) | 6617 (64.3%) | **<0.001** | 582 (89.7%) | 6617 (64.3%) | **<0.001** | 696 (93.9%) | 6617 (64.3%) | **<0.001** | 676 (91.5%) | 6617 (64.3%) | **<0.001** |
| Duration of infertility (y) | 3.3±2.2 | 3.1±2.2 | **<0.001** | 3.1±2.4 | 3.1±2.2 | 0.90 | 2.9±2.3 | 2.9±2.3 | **0.001** | 3.4±2.4 | 3.1±2.2 | **0.004** |
| **Paternal characteristic** |  |  |  |  |  |  |  |  |  |  |  |  |
| Age (y) | 32.7±4.3 | 32.5±4.1 | **0.03** | 31.4±4.1 | 32.5±4.1 | **<0.001** | 30.9±4.2 | 32.5±4.1 | **<0.001** | 30.9±3.9 | 32.5±4.1 | **<0.001** |
| Frequent alcohol  intake, No. (%) | 49 (1.9%) | 254 (2.5%) | 0.09 | 19 (2.9%) | 254 (2.5%) | 0.47 | 15 (2.0%) | 254 (2.5%) | 0.45 | 14 (1.9%) | 254 (2.5%) | 0.33 |
| Tobacco use, No. (%) | 695 (26.9%) | 3270 (31.8%) | **<0.001** | 153 (23.6%) | 3270 (31.8%) | **<0.001** | 187 (25.2%) | 3270 (31.8%) | **<0.001** | 229 (31.0%) | 3270 (31.8%) | 0.65 |
| Higher education, No. (%) | 1362 (52.7%) | 5540 (53.9%) | 0.28 | 355 (54.7%) | 5540 (53.9%) | 0.68 | 400 (54.0%) | 5540 (53.9%) | 0.96 | 340 (46.0%) | 5540 (53.9%) | **<0.001** |
| Parotitis, No. (%) | 113 (4.4%) | 300 (2.9%) | **<0.001** | 58 (8.9%) | 300 (2.9%) | **<0.001** | 110 (14.8%) | 300 (2.9%) | **<0.001** | 77 (10.4%) | 300 (2.9%) | **<0.001** |
| **IVF/ICSI characteristics** |  |  |  |  |  |  |  |  |  |  |  |  |
| Ovarian stimulation protocol |  |  | 0.08 |  |  | **0.001** |  |  | 0.54 |  |  | **<0.001** |
| Agonist | 1682 (65.1%) | 6498 (63.2%) |  | 453 (69.8%) | 6498 (63.2%) |  | 460 (62.1%) | 6498 (63.2%) |  | 419 (56.7%) | 6498 (63.2%) |  |
| Antagonist | 903 (34.9%) | 3785 (36.8%) |  | 196 (30.2%) | 3785 (36.8%) |  | 281 (37.9%) | 3785 (36.8%) |  | 320 (43.3%) | 3785 (36.8%) |  |
| Gn duration (days) | 10.2±1.8 | 10.1±1.8 | 0.71 | 10.3±1.8 | 10.1±1.8 | 0.06 | 10.2±1.8 | 10.1±1.8 | 0.85 | 10.1±1.8 | 10.1±1.8 | 0.21 |
| Total dose of Gn (IU) | 2287.6±814.4 | 2310.5±825.1 | 0.21 | 2310.5±825.1 | 2310.5±825.1 | **0.04** | 2243.9±793.0 | 2310.5±825.1 | **0.03** | 2210.5±796.0 | 2310.5±825.1 | **0.001** |
| Estradiol on trigger day (pg/mL) | 2784.8±1696.0 | 2787.4±1656.4 | 0.94 | 2956.0±1753.0 | 2787.4±1656.4 | **0.01** | 2901.5±1586.4 | 2376 (1855) | 0.07 | 2956.7±1612.9 | 2787.3±1656.4 | **0.01** |
| Fertilisation method (n) |  |  | **<0.001** |  |  | **<0.001** |  |  | **<0.001** |  |  | **<0.001** |
| IVF | 1039 (40.2%) | 8068 (78.5%) |  | 0 (0.0%) | 8068 (78.5%) |  | 0 (0.0%) | 8068 (78.5%) |  | 635 (85.9%) | 8068 (78.5%) |  |
| ICSI | 1546 (59.8%) | 2215 (21.5%) |  | 649 (100.0%) | 2215 (21.5%) |  | 741 (100.0%) | 2215 (21.5%) |  | 104 (14.1%) | 2215 (21.5%) |  |
| Endometrial thickness (mm) | 11.6±3.3 | 11.4±3.3 | **<0.001** | 12.2±2.6 | 11.5±2.6 | **<0.001** | 12.1±2.5 | 11.5±2.6 | **<0.001** | 11.9±3.2 | 11.4±3.3 | **<0.001** |
| Number of oocytes  retrieved (n) | 13.6±6.6 | 13.6±6.5 | 0.82 | 14.5±6.9 | 14.0±6.5 | **<0.001** | 14.6±6.4 | 13.6±6.5 | **<0.001** | 14.5±6.7 | 13.6±6.5 | **<0.001** |

^a^ PSM factors included female age, female BMI, male age, male BMI, ovarian stimulation protocol, number of oocytes retrieved, and endometrial thickness

Data are presented as mean±SD or proportions (percentage). Data were compared using Student’s t-test for continuous variables and chi-square test for categorical variables. *P*-values in bold are significant (*P* < 0.05).

PSM, propensity score matching; IVF, *in-vitro* fertilisation; ICSI, intracytoplasmic sperm injection; MMF, mild-moderate male factor; OAT-S, severe oligoasthenoteratozoospermia; Azoospermia-H, Azoospermia-husband; Azoospermia-D, Azoospermia-donor; FSH, follicle stimulating hormone; AMH, anti-Müllerian Hormone; BMI, body mass index; Gn: gonadotropins.

**Supplementary Table S2.** Baseline Characteristics After 1:1 PSM for Fertilisation Comparison

| **Variable** | **MMF** | **Control ^a^** | ***P*** | **OAT-S** | **Control ^a^** | ***P*** | **Azoospermia-H** | **Control ^a^** | ***P*** | **Azoospermia-D** | **Control ^a^** | ***P*** |
| --- | --- | --- | --- | --- | --- | --- | --- | --- | --- | --- | --- | --- |
| No. of cycles | 1038 | 1038 |  | 635 | 635 |  | 698 | 698 |  | 622 | 622 |  |
| Female age (y) | 31.3±3.8 | 31.2±3.6 | 0.53 | 29.5±3.6 | 29.5±3.6 | >0.99 | 29.3±3.5 | 29.2±3.6 | 0.97 | 29.0±3.5 | 29.0±3.5 | 0.80 |
| BMI (kg/m^2^) | 21.8±2.8 | 21.8±2,7 | 0.61 | 21.8±2.9 | 21.8±2.8 | 0.86 | 21.7±2.9 | 21.7±2.7 | 0.92 | 21.6±2.9 | 21.7±2.9 | 0.55 |
| Male age (y) | 32.9±4,2 | 32.9±4.1 | 0.84 | 31.5±4.1 | 31.4±4.0 | 0.97 | 31.1±4.1 | 31.1±3.8 | 0.91 | 31.0±3.9 | 31.0±4.1 | 0.72 |
| Ovarian stimulation protocol |  |  | 0.41 |  |  | 0.72 |  |  | 0.91 |  |  | 0.57 |
| Agonist | 675 (65.0%) | 693 (66.8%) |  | 439 (69.1%) | 433 (68.2%) |  | 445 (63.8%) | 443 (63.5%) |  | 338 (54.3%) | 348 (55.9%) |  |
| Antagonist | 363 (35.0%) | 345 (33.2%) |  | 196 (30.9%) | 202 (31.8%) |  | 253 (36.2%) | 255 (36.5%) |  | 284 (45.7%) | 274 (44.1%) |  |
| Gn time (days) | 10.1±1.7 | 10.2±1.9 | 0.08 | 10.3±1.7 | 10.2±1.8 | 0.39 | 10.2±1.8 | 10.1±1.8 | 0.22 | 10.0±1.8 | 10.0±1.8 | 0.94 |
| Gn dose (IU) | 2318.3±809.8 | 2336.3±828.5 | 0.62 | 2253.2±828.6 | 2185.2±802.0 | 0.14 | 2215.3±732.5 | 2164.6±783.6 | 0.21 | 2174.4±730.4 | 2128.7±816.5 | 0.30 |
| No. of oocytes | 13,1±6.5 | 13.2±6.4 | 0.71 | 14.5±7.0 | 14.7±6.7 | 0.73 | 14.6±6,4 | 14.7±7.0 | 0.88 | 14.5±6.6 | 14.8±6.9 | 0.54 |
| No. of MII | 11.5±5.8 | 11.6±5.7 | 0.50 | 11.8±5.9 | 12.0±5.8 | 0.64 | 11.8±5.3 | 11.9±5.9 | 0.88 | 12.8±6.0 | 12.9±6.2 | 0.68 |

^a^ PSM factors included female age, female BMI, male age, male BMI, ovarian stimulation protocol, and number of oocytes retrieved.

Data are presented as mean±SD or proportions (percentage). Data were compared using Student’s t-test for continuous variables and chi-square test for categorical variables.

PSM, propensity score matching; MMF, mild-moderate male factor; OAT-S, severe oligoasthenozoospermia; Azoospermia-H, azoospermia-husband; Azoospermia-D, Azoospermia-donor; BMI, body mass index; COH, controlled ovarian hyperstimulation; Gn: gonadotropins; MII, metaphase II.

**Supplementary Table S3. Analysis of additional groups：**Embryologic outcomes after PSM.

| **Variable** | **NOA** | **Control ^a^** | **β (95% CI)** | **OA** | **Control ^a^** | **β (95% CI)** | **Teratosper**  **mia** | **Control ^a^** | **β (95% CI)** | **Cryptozoos**  **permia** | **Control ^b^** | **β (95% CI)** |  |
| --- | --- | --- | --- | --- | --- | --- | --- | --- | --- | --- | --- | --- | --- |
| No. of cycles | 110 | 438 |  | 609 | 2302 |  | 574 | 2287 |  | 31 | 121 |  |  |
| No. of MII (n) | 11 (7, 14) | 11 (8, 15) | -0.215  (-1.368, 0.938) | 12 (9, 16) | 12 (8, 16) | -0.006  (-0.520, 0.508) | 11 (8, 15) | 11 (8,15) | 0.124  (-0.383, 0.631) | 11 (9, 16) | 12 (9, 17) | -0.276  (-2.259, 1.706) |  |
|  | 10.9±5.4 | 11.1±5.6 |  | 11.9±5.3 | 12.0±5.9 |  | 11.2±5.8 | 11.1±5.5 |  | 12.5±5.3 | 12.7±4.8 |  |  |
| No. of 2PN (n) | 6 (4, 11) | 8 (5, 11) | **-1.589**  **(-2.515, -0.664)** | 8 (5, 11) | 8 (6, 12) | **-0.550**  **(-0.963, -0.136)** | 8 (6, 11) | 8 (6, 11) | 0.173  (-0.236, 0.581) | 8 (5, 11) | 9 (7, 12) | -0.148  (-2.943, 0.247) |  |
|  | 6.3±4.2 | 7.9±4.5 |  | 8.0±4.3 | 8.5±4.7 |  | 8.2±4.6 | 8.0±4.4 |  | 8.0±3.8 | 9.6±3.9 |  |  |
| Normal fertilisation rate (%)^c^ | 58.7±25.1 | 72.9±17.8 | **-0.142**  **(-0.184, -0.100)** | 66.3±20.0 | 72.2±18.8 | **-0.059**  **(-0.081, -0.037)** | 73.8±19.4 | 72.2±18.1 | 0.016  (-0.022,0.054) | 65.0±24.2 | 72.2±16.4 | -0.062  (-0.137, 0.013) |  |
| No. of normal cleavage embryos (n) | 6 (4, 11) | 8 (5, 11) | **-1.738**  **(-2.663, -0.813)** | 8 (5, 11) | 8 (6, 12) | **-0.740**  **(-1.161, -0.318)** | 8 (5, 11) | 8 (5, 11) | -0.063  (-0.478, 0.353) | 8 (5, 11) | 9 (7, 11) | -1.791  (-3.422, 0.160) |  |
|  | 6.2±4.1 | 8.0±4.5 |  | 8.0±4.2 | 8.7±4.8 |  | 8.1±4.5 | 8.2±4.5 |  | 7.9±3.8 | 9.4±3.8 |  |  |
| Normal cleavage rate (%) | 97.6±7.1 | 98.0±6.2 | -0.005  (-0.018, 0.009) | 98.6±44.5 | 98.1±59.5 | 0.005  (0.000, 0.010) | 98.0±67.5 | 98.1±65.2 | 0.000  (-0.007, 0.006) | 96.9±61.5 | 98.2±4.4 | -0.014  (-0.033, 0.004) |  |
| No. of D3 high-quality cleavage embryos (n) | 3 (1, 5) | 4 (2, 6) | **-1.011**  **(-0.305, -1.716)** | 4 (2, 6) | 4 (2, 6) | -0.167  (-0.513, 0.178) | 4 (2, 6) | 4 (2, 6) | -0.015  (-0.309, 0.280) | 4 (1, 7) | 5 (2, 7) | -0.834  (-2.231, 0.563) |  |
|  | 2.9±2.6 | 3.9±3.0 |  | 4.1±3.1 | 4.3±3.3 |  | 4.0±3.0 | 4.1±3.0 |  | 3.8±3.3 | 4.8±2.9 |  |  |
| D3 high-quality cleavage embryo rate (%) | 40.3±24.2 | 49.2±27.0 | **-0.089**  **(-0.154, -0.025)** | 49.2±26.5 | 49.2±26.2 | 0.001  (-0.027, 0.028) | 49.3±26.2 | 49.7±26.7 | -0.004  (-0.030, 0.022) | 47.6±34.9 | 50.2±25.2 | -0.037  (-0.17, 0.099) |  |
| No. of total cryopreserved blastocyst (n) | 2 (1, 4) | 3 (2, 5) | **-0.950**  **(-1.537, -0.362)** | 3 (1, 5) | 3 (2, 6) | **-0.495**  **(-0.774, -0.216)** | 3 (2, 6) | 3 (2, 6) | 0.124  (-0.152, 0.400) | 3 (1, 4) | 4 (2, 7) | -1.422  (-2.548, 0.296) |  |
|  | 2.3±2.6 | 3.3±2.9 |  | 3.1±2.9 | 3.6±3.2 |  | 3.5±3.1 | 3.3±3.0 |  | 2.7±2.3 | 4.0±2.9 |  |  |
| Blastocyst formation rate (%) | 57.8±31.9 | 68.9±30.8 | **-0.111**  **(-0.174, -0.048)** | 62.5±29.8 | 68.4±26.9 | **-0.059**  **(-0.084, -0.033)** | 70.0±26.1 | 70.0±26.7 | 0.003  (-0.022, 0.029) | 60.4±33.3 | 71.4±26.7 | -0.116  (-0.228, 0.004) |  |
| Viable blastocyst formation rate (%) | 42.8±29.1 | 47.3±26.6 | -0.045  (-0.106, 0.016) | 42.6±28.0 | 46.3±26.5 | **-0.037**  **(-0.062, -0.012)** | 46.5±27.4 | 47.0±27.2 | -0.005  (-0.031, 0.021) | 39.4±32.5 | 48.2±25.0 | -0.087  (-0.196, 0.022) |  |
| No. of available embryos (n) | 3 (2, 5) | 4 (3, 6) | **-0.997**  **(-1.543, -0.450)** | 4 (2, 6) | 5 (3, 7) | **-0.492**  **(-0.751, -0.233)** | 4 (3, 7) | 4 (3, 7) | 0.097  (-0.159, 0.353) | 4 (3, 6) | 5 (3, 7) | -1.326  (-2.390, 0.261) |  |
|  | 3.4±2.5 | 4.4±2.7 |  | 4.2±2.7 | 4.6±2.9 |  | 4.6±2.9 | 4.5±2.8 |  | 3.8±2.4 | 4.9±2.8 |  |  |
| Embryo utilisation index (%) | | 58.3±28.5 | 60.6±24.2 | -0.023  (-0.075, 0.030) | 54.2±29.4 | 57.6±23.7 | **-0.034**  **(-0.056, -0.013)** | 58.4±24.4 | 59.3±24.3 | -0.009  (-0.031, 0.013) | 47.6±27.7 | 55.7±22.2 | -0.098  (-0.195, 0.001) |

^a^ PSM factors included female age, female BMI, male age, male BMI, COH protocol, number of oocytes retrieved, and endometrial thickness.

^b^ PSM factors included female age, number of oocytes retrieved, and male age.

^c^ Fertilisation method (IVF or ICSI) were chosen from control group. Baseline characteristics of patients were matched by 1:1 PSM. PSM factors of Cryptozoospermia group included female age, number of oocytes retrieved, and male age. PSM factors of NOA, OA, Teratospermia groups included female age, female BMI, male age, male BMI, COH protocol, and number of oocytes retrieved.

Data are presented as mean ± SD or median (Q25, Q75). Data were compared using the univariate linear regression analysis. Bold values indicate statistically significant differences.

PSM, propensity score matching; NOA, non-obstructive azoospermia; OA, obstructive azoospermia; β (95% CI), β-coefficient with 95% confidence interval; MII, metaphase II; PN, Pronucleus.

**Supplementary Table S4. Analysis of additional groups：**Pregnancy and obstetric and neonatal outcomes after PSM.

| **Variable** | **NOA** | **Control ^a^** | **OR (95% CI)** | **OA** | **Control ^a^** | **OR (95% CI)** | **Teratospermia** | **Control ^a^** | **OR (95% CI)** | **Cryptozoospermia** | **Control ^b^** | **OR (95% CI)** |
| --- | --- | --- | --- | --- | --- | --- | --- | --- | --- | --- | --- | --- |
| No. of cycles | 110 | 438 |  | 609 | 2302 |  | 574 | 2287 |  | 31 | 121 |  |
| **Pregnancy outcomes** |  |  |  |  |  |  |  |  |  |  |  |  |
| Cumulative  clinical pregnancy | 82 (74.5%) | 354 (80.8%) | 0.70  (0.43, 1.14) | 492 (80.8%) | 1854 (80.5%) | 1.02  (0.81, 1.27) | 439 (76.5%) | 1827 (79.9%) | 0.82  (0.66, 1.02) | 19 (61.3%) | 98 (81.0%) | **0.37**  **(0.16, 0.87)** |
| Ectopic pregnancy | 1 (0.9%) | 5 (1.1%) | 0.79  (0.09, 6.87) | 1 (0.2%) | 23 (1.0%) | 0.16  (0.02,1. 21) | 4 (0.7%) | 23 (1.0%) | 0.69  (0.24, 2.01) | 0 (0.0%) | 1 (0.8%) | NA |
| Pregnancy loss | 20 (18.2%) | 41 (9.4%) | **2.15**  **(1.20, 3.85)** | 77 (12.6%) | 307 (13.3%) | 0.94  (0.72, 1.23) | 55 (9.6%) | 267 (11.7%) | 0.80  (0.59, 1.09) | 0 (0.0%) | 21 (17.4%) | NA |
| Cumulative  live births | 73 (66.4%) | 332 (75.8%) | **0.63**  **(0.40, 0.99)** | 460 (75.5%) | 1677 (72.8%) | 1.15  (0.94, 1.41) | 402 (70.0%) | 1664 (72.8%) | 0.88  (0.72, 1.07) | 19 (61.3%) | 85 (70.2%) | 0.67  (0.30, 1.52) |
| **Obstetric and neonatal**  **outcomes in singleton** |  |  |  |  |  |  |  |  |  |  |  |  |
| Preterm birth | 4/71  (5.6%) | 21/306  (6.9%) | 0.81  (0.27, 2.44) | 28/435  (6.4%) | 106/1569 (6.8%) | 0.95  (0.62, 1.46) | 22/376  (5.9%) | 133/1560  (8.5%) | 0.67  (0.42, 1.06) | 2/18  (11.1%) | 8/80  (10.0%) | 1.13  (0.22, 5.81) |
| Hypertensive disorders  of pregnancy | 3/71  (4.2%) | 6/306  (2.0%) | 2.21  (0.54, 9.04) | 5/435  (1.1%) | 14/1569  (0.9%) | 1.29  (0.46, 3.61) | 8/376  (2.1%) | 35/1560  (2.2%) | 0.95  (0.44, 2.06) | 1/18  (5.6%) | 3/80  (3.8%) | 1.51  (0.15, 15.41) |
| Gestational diabetes  mellitus | 2/71  (2.8%) | 5/306  (1.6%) | 1.75  (0.33, 9.18) | 7/435  (1.6%) | 40/1569  (2.5%) | 0.63  (0.28, 1.41) | 3/376  (0.8%) | 33/1560  (2.1%) | 0.37  (0.11, 1.22) | 0/18  (0.0%) | 3/80  (3.8%) | NA |
| Placenta previa | 3/71  (4.2%) | 10/306  (3.3%) | 1.31  (0.35, 4.87) | 10/435  (2.3%) | 33/1569  (2.1%) | 1.10  (0.54, 2.24) | 5/376  (1.3%) | 38/1560  (2.4%) | 0.54  (0.21, 1.38) | 1/18  (5.6%) | 2/80  (2.5%) | 2.29  (0.20, 26.78) |
| Foetal malformation | 1/71  (1.4%) | 5/306  (1.6%) | 0.86  (0.10, 7.48) | 4/435  (0.9%) | 22/1569 (1.4%) | 0.65  (0.22, 1.90) | 1/376  (0.3%) | 17/1560  (1.1%) | 0.24  (0.03, 1.82) | 0/18  (0.0%) | 1/80  (1.3%) | NA |
| Placenta abruption | 1/71  (1.4%) | 0/306  (0.0%) | NA | 0/435  (0.0%) | 2/1569  (0.1%) | NA | 0/376  (0.0%) | 2/1560  (0.1%) | NA | 0/18  (0.0%) | 0/80  (0.0%) | NA |
| Macrosomia  ＞4000g | 6/71  (8.5%) | 11/306  (3.6%) | 2.48  (0.88, 6.94) | 20/435  (4.6%) | 76/1569 (4.8%) | 0.95  (0.57, 1.57) | 15/376  (4.0%) | 59/1560  (3.8%) | 1.06  (0.59, 1.89) | 0/18  (0.0%) | 3/80  (3.8%) | NA |
| Low birthweight  ＜2500g | 5/71  (7.0%) | 16/306  (5.2%) | 1.37  (0.49,3.88 ) | 18/435  (4.1%) | 53/1569 (3.4%) | 1.24  (0.72, 2.13) | 11/376  (2.9%) | 69/1560  (4.4%) | 0.65  (0.34, 1.24) | 1/18  (5.6%) | 5/80  (6.3%) | 0.88  (0.10, 8.05) |

^a^ PSM factors included female age, female BMI, male age, male BMI, COH protocol, number of oocytes retrieved, and endometrial thickness.

^b^ PSM factors included female age, number of oocytes retrieved, and male age.

Data are presented as proportions (percentage). Odds ratio (OR) and 95% confidence intervals (CIs) are based on the univariate logistic regression analysis. Bold values indicate statistically significant differences.

NA, not applicable (no cases, therefore odds ratio not estimable).

PSM, propensity score matching; NOA, non-obstructive azoospermia; OA, obstructive azoospermia.
